# Indices of sarcopenic obesity are important predictors of finite element analysis-derived bone strength in older adults with obesity

**DOI:** 10.3389/fendo.2023.1279321

**Published:** 2023-11-07

**Authors:** Giulia Gregori, Arjun Paudyal, Yoann Barnouin, Alessandra Celli, Martha Belen Segoviano-Escobar, Reina Armamento-Villareal, Nicola Napoli, Clifford Qualls, Dennis T. Villareal

**Affiliations:** ^1^ Division of Endocrinology, Diabetes, and Metabolism, Baylor College of Medicine, Houston, TX, United States; ^2^ Center for Translational Research on Inflammatory Diseases, Michael E DeBakey Veterans Affairs (VA) Medical Center, Houston, TX, United States; ^3^ Divison of Bone and Mineral Diseases, Washington University School of Medicine, St Louis, MO, United States; ^4^ Operative Research Unit of Osteo-metabolic Diseases, Fondazione Policlinico Universitario Campus Bio-Medico, Roma, Italy; ^5^ Department of Mathematics and Statistics, School of Medicine, University of New Mexico, Albuquerque, NM, United States

**Keywords:** adiposity, aging, sarcopenia, bone quality, body composition, muscle strength

## Abstract

**Background:**

The expanding population of older adults with obesity is a public health challenge, in part, because of the increased risk of fractures despite normal or high bone mineral density. Potential factors predisposing to fractures in this group include sarcopenia associated with obesity and impaired bone quality. We aimed to determine the contribution of sarcopenic obesity (SO) indices to bone strength as assessed by microfinite element analysis (μFEA) of high-resolution peripheral quantitative computed tomography (HR-pQCT).

**Methods:**

One-hundred eighty-nine older (age ≥ 65 years) adults with obesity (BMI ≥ 30 kg/m^2^) participated in lifestyle intervention trials at our medical center. All underwent baseline measurements of bone strength (failure load and stiffness) using μFEA from HR-pQCT of the distal radius and tibia. In addition, SO indices [appendicular lean mass/weight (ALM/W) and percent body fat (FM%)] by dual-energy X-ray absorptiometry and handgrip strength (HGS) by dynamometry were assessed. SO was diagnosed and staged based on the 2022 ESPEN and EASO expert consensus statement.

**Results:**

Both ALM/W and HGS were positively correlated explaining 28% to 36% of the variance in failure load and stiffness at the distal radius and tibia (all *p* < 0.001). In contrast, FM% was negatively correlated explaining 22% to 31% of the variance in failure load and stiffness at the distal radius and tibia (all *p* < 0.001). The associations of SO indices with failure load and stiffness remained significant after controlling for age, sex, race/ethnicity, diabetes, and 25-OH vitamin D (ALM/W: *R* ^2 = ^0.301 to 0.448, HGS: *R* ^2 = ^0.346 to 0.472, FM%: *R* ^2 = ^0.299 to 0.432) (*p* < 0.001 to 0.011). SO was diagnosed in 75/189 (40%) participants with 66/75 (88%) having functional or metabolic complications (stage II). Participants with SO had lower failure load and stiffness at the distal radius than participants with no SO (both *p* < 0.05).

**Conclusion:**

These findings demonstrate that lower muscle mass and strength and higher fat mass may impair bone quality. Therefore, interventions that focus on preserving muscle mass and strength while reducing fat mass may be important to decrease fracture risk when older adults with obesity undertake lifestyle intervention therapy.

## Introduction

The number of older adults with obesity worldwide is rapidly expanding because of both an increase in the total number of older adults (age ≥ 65 years) and in the percentage of the older population who are obese [body mass index (BMI) ≥ 30 kg/m^2^] (1, 2). In older adults, obesity increases the risk of fractures especially at the ankle and humerus despite normal or high bone mineral density (BMD) (3, 4). Potential factors predisposing to fractures in this population are sarcopenia (low muscle mass relative to body weight) and impaired bone quality (low bone strength independent of BMD) (5, 6). Indeed, obesity causes sarcopenic obesity (SO) (coexistence of low muscle mass/strength and excess adiposity) in older adults (7), which has been associated with an increased incidence of fractures (8). In addition to mechanical loading of the skeleton (9), the muscle may positively impact bone strength by releasing myokines (10), while fat may negatively impact bone strength by releasing adipokines (11) within the bone microenvironment (“muscle–fat–bone interactions”) (12). However, little is known about how the adverse age- and obesity-related changes in muscle mass and fat mass contribute to bone strength in older adults with obesity. This information may be important for developing optimal strategies for preserving bone strength during weight loss therapy of older adults with obesity (13).

Common tools for diagnosing osteoporosis such as dual-energy X-ray absorptiometry (DXA) that measures areal BMD (aBMD) can be subject to various scanning artifacts (e.g., from osteophytes or aortic calcifications in older individuals and thickness of overlying fat in obese individuals) (14, 15). Moreover, such tools usually provide information only on bone quantity, not bone quality, an important determinant of fracture (16). Bone quality refers to the material and structural properties of bone strength (16). Microfinite element analysis (μFEA) of high-resolution peripheral quantitative computed tomography (HR-pQCT) is a novel way of assessing bone strength by quantitively deriving biochemical properties resulting from high-resolution 3D images of bone microarchitecture (17). The aim of this study was to determine the contribution of indices used to diagnose SO [appendicular lean mass/weight (ALM/W), percent body fat (FM%), and hand grip strength (HGS)] (18) to bone quality as assessed by μFEA of bone strength (failure load and stiffness) at the distal radius and tibia.

In this population of older adults with obesity, we hypothesized that ALM/W and HGS would positively contribute while FM% would negatively contribute to failure load and stiffness at the distal radius and tibia. We also hypothesized that those meeting the criteria for SO (18) would have a higher prevalence of functional or metabolic complications.

## Methods

### Study population

The current study is a secondary analysis of baseline data from older adults with obesity consecutively recruited to two lifestyle intervention trials (diet-induced weight loss and exercise training) at the Michael E DeBakey VA Medical Center (MEDVAMC) in Houston, TX (NCT03329963 and NCT02367105). Participants were recruited from the community through advertisements. Persons were eligible for inclusion in the trials if they were 65 years of age or older, were obese (BMI ≥ 30 kg/m^2^), were sedentary (regular exercise <1 h/week), and had had a stable body weight ( ± 2 kg) and stable medication use for 6 months prior to enrolment. Persons who had severe cardiopulmonary disease (e.g., recent myocardial infarction, unstable angina), musculoskeletal impairments that precluded exercise training, cognitive impairments (Mini Mental State Exam score < 24), and osteoporosis (*T*-score −2.5 and below or history of fragility fractures) or who used drugs that affect bone metabolism (e.g., bisphosphonates, glucocorticoids) or had secondary osteoporosis (e.g., rheumatoid arthritis) were excluded from participation. The current study was approved by the Institutional Review Board of Baylor College of Medicine and the Research and Development Committee of the MEDVAMC.

### Anthropometrics, blood pressure, and fasting blood samples

Body weight was measured in the morning without shoes following a 12-h fast. Height was measured to the nearest 0.5 cm and BMI was calculated. Waist circumference was measured horizontally at the midpoint between the highest point of the iliac crest and the lowest portion of the 12^th^ rib. Blood pressure (BP) was measured with a sphygmomanometer cuff of the appropriate size after the subject rested for approximately 5 min. Serum lipoprotein levels were measured by using an automated enzymatic/colorimetric assay (Roche/Hitachi System, Indianapolis, IN, USA). Plasma glucose was determined by using the glucose oxidase method (YSI Stat Plus; YSI, Yellow Springs, OH, USA). 25-Hydroxy vitamin D (25-OH vitamin D) was measured using the immunochemiluminometric assay (Diasorin Liaison, Chicago, IL, USA).

### Body composition and bone mineral density

Lean mass and fat mass of the total body, arms (both right and left), and legs (both right and left) were measured by using dual-energy X-ray absorptiometry (DXA; Hologic Horizon APEX Software 5.5.2). Appendicular body composition was calculated by summing the right and left arms and legs. Areal BMD (aBMD) of the total body, hip, and lumbar spine was also measured by using DXA. The CV at our center for body composition is 1.5% (19), and for hip and lumbar spine, it is 1.2% (20).

### Handgrip strength

HGS was measured by using a JAMAR handheld dynamometer (Sammons Preston Rolyan, Bolingbrook, IL, USA) following a standard protocol. Briefly, the test was performed in the seated position, with the shoulder adducted and neutrally rotated, the elbow flexed to 90°, and the forearm and wrist in a neutral position. Participants were instructed to squeeze the handle as hard as possible for approximately 3 s. This was repeated three times alternating between right and left hands and 30 s rest between trials. The best of the six grip strength measurements was used for analysis.

### Bone strength

Bone microarchitecture and mechanical properties were measured by using HR-pQCT (Xtreme CTII, Scanco Medical AG, Brüttisellen, Switzerland) at the non-dominant distal radius and tibia as previously described by our group (21). In brief, a scout view was performed, and a reference line was placed at the endplate of the radius or tibia. Then, the first slide was acquired at 9.0 and 22.0 mm proximal to the bone of interest. The mineralized bone phase was extracted by using a low-pass Gaussian filer. Bone was extracted with a fixed threshold of 320 mg HA/cm^3^ for trabecular and 450 mg HA/cm^3^ for cortical. Bone microarchitecture was assessed in the trabecular and cortical regions by using voxel-based measurements. Segmentation between cortical and trabecular bone was done when necessary. μFEA element analysis of the radius and tibia, represented by failure load and stiffness, was performed creating μFEA models that accurately describe the actual bone microarchitecture in detail, automatically converting each voxel of bone tissue into equally sized brick elements, as previously described (22). Cortical and trabecular bone elements were assigned a Young’s modulus of 20 and 17 GPa, respectively, and a Poisson’s ratio (23). The μFEA consists of a compression test simulation in which a load in the longitudinal direction is applied at one end, while the other end is fully constrained, to simulate a fall from standing height on an outstretched hand (24). The failure load was calculated by using the criterion developed by Pistoia et al., which has been shown to predict failure load measured by loading cadaver forearms (24). Stiffness is the extent to which an object resists deformation in response to an applied force. The same parameters were used for the tibia analysis, as this was shown earlier to be associated with fragility fractures. The CV for μFEA parameters of HR-pQCT is 2.0% to 3.5% (25).

### Diagnosis and staging of sarcopenic obesity

The diagnosis of SO was based on the 2022 European Society for Clinical Nutrition and Metabolism (ESPEN) and the European Association for the Study of Obesity (EASO) international expert consensus statement (18). Accordingly, participants who had altered skeletal muscle function (HGS < 35.5 kg for men and <20.0 kg for women) (26) and altered body composition (FM > 25% for men and >32% for women and ALM/W < 25.7% for men (27) and <19.4% for women (28)) met the criteria for SO. Participants with no complications were considered to have stage I, while those with at least one complication attributable to SO were considered to have stage II (18). For this study, the presence of functional or metabolic complications was operationally defined as having physical frailty or cardiometabolic syndrome, respectively. Participants who had a score of <31 on the Physical Performance Test (PPT: range from 0 to 36, with higher scores indicating better physical function) were defined as having evidence of physical frailty (29, 30). Briefly, the PPT includes seven standardized tasks (walking 50 ft, putting on and removing a coat, picking up a penny, standing up from a chair, lifting a book, climbing one flight of stairs, and performing a progressive Romberg test) plus two additional tasks (climbing up and down four flights of stairs and performing a 360° turn). Participants who met ≥3 of the following criteria (31) were defined as having cardiometabolic syndrome: waist circumference ≥102 cm for men and ≥88 cm for women, triglycerides ≥150 mg/dL or drug treatment for elevated triglycerides, HDL cholesterol <40 mg/dL for men and <50 mg/dL for women, systolic BP ≥130 mmHg or diastolic BP ≥85 mmHg or on antihypertensive treatment with a history of hypertension, and fasting plasma glucose ≥110 mg/dL or on glucose-lowering agent with history of diabetes.

### Statistical analysis

Statistical Analysis Software (SAS Institute Inc, Version 9.4, Carey, NC, USA) was used for all statistical analyses. Descriptive estimates of body composition and SO indices were stratified by sex. Bivariate and multivariable linear regression analyses assessed the associations between SO indices (ALMI/W, FM%, and HGS) and bone strength (failure load and stiffness) at the distal radius and tibia. Since there were no significant SO indices × sex interaction effects, sex was entered as a covariate. In addition to sex, age, race/ethnicity, diabetes, and 25-OH vitamin D were entered in the full model due to their established effects on body composition and bone strength (32, 33). For multivariable regression analyses, multicollinearity was assessed by using the variance inflation factor (VIF). Since ALMI/W, FM%, and HGS were highly correlated (*R* = −0.93 to 0.65; *p* < 0.001) and the combined multivariable models regressing ALM/W, FM%, and HGS on fracture load and stiffness adjusted for confounders resulted in VIF >5.0, each SO index was studied separately by multivariable regression models. Standardized beta values (*β*) and standard errors for standardized *β* are presented. Statistical significance of the *p*-values in the multivariable regression models was controlled for multiple testing with the Benjamini–Hochberg procedure (34). The proportions of participants with physical frailty and cardiometabolic syndrome were compared between the groups with SO and no SO using Fisher’s exact test. Differences in bone strength and aBMD were compared between the groups with SO and no SO using analyses of covariance with age, sex, race/ethnicity, diabetes, and 25-OH vitamin D as covariates. *p*-values <0.05 were considered to indicate statistical significance.

## Results

Participants were older adults with obesity (**Table 1**). Approximately two-thirds were of male sex and white race, and the majority were well educated. Consistent with an at-risk population, participants had a high prevalence of not only physical frailty but also cardiometabolic syndrome associated with chronic medication use.

**Table 1:** Characteristics of the study participants (*n* = 189).

Age (years)	71.0 ± 4.2
Weight (kg)	106.2 ± 18.1
Height (cm)	170.8 ± 9.4
Body mass index (kg/m^2^)	36.3 ± 4.7
Sex, *n* (%)	
Male	128 (68)
Female	61 (32)
Race/ethnicity, *n* (%)	
White	108 (57)
Black	45 (24)
Hispanic	25 (13)
Other	11 (6)
Education, *n* (%)	
Less than college degree	37 (20)
College degree	78 (41)
Graduate school	74 (39)
Physical frailty, *n* (%)	121 (64)
Cardiometabolic syndrome, *n* (%)	142 (75)
Physical Performance Test (score)	30.5 ± 3.2
Diabetes, *n* (%)	84 (44%)
Systolic blood pressure (mmHg)	133. ± 14.9
Diastolic blood pressure (mmHg)	81.3 ± 8.2
Waist circumference (cm)	119.7 ± 12.7
Triglyceride (mg/dL)	118.2 ± 55.5
HDL-cholesterol (mg/dL)	48.1 ± 13.8
Glucose (mg/dL)	106.9 ± 34.5
25-OH vitamin D (ng/mL)	35.1 ± 14.3
Medication use, *n* (%)	
Antihypertensive	137 (73)
Antilipidemic	98 (52)
Antidiabetes	57 (30)

Values are means ± SD or n (%).

**Figure 1:**
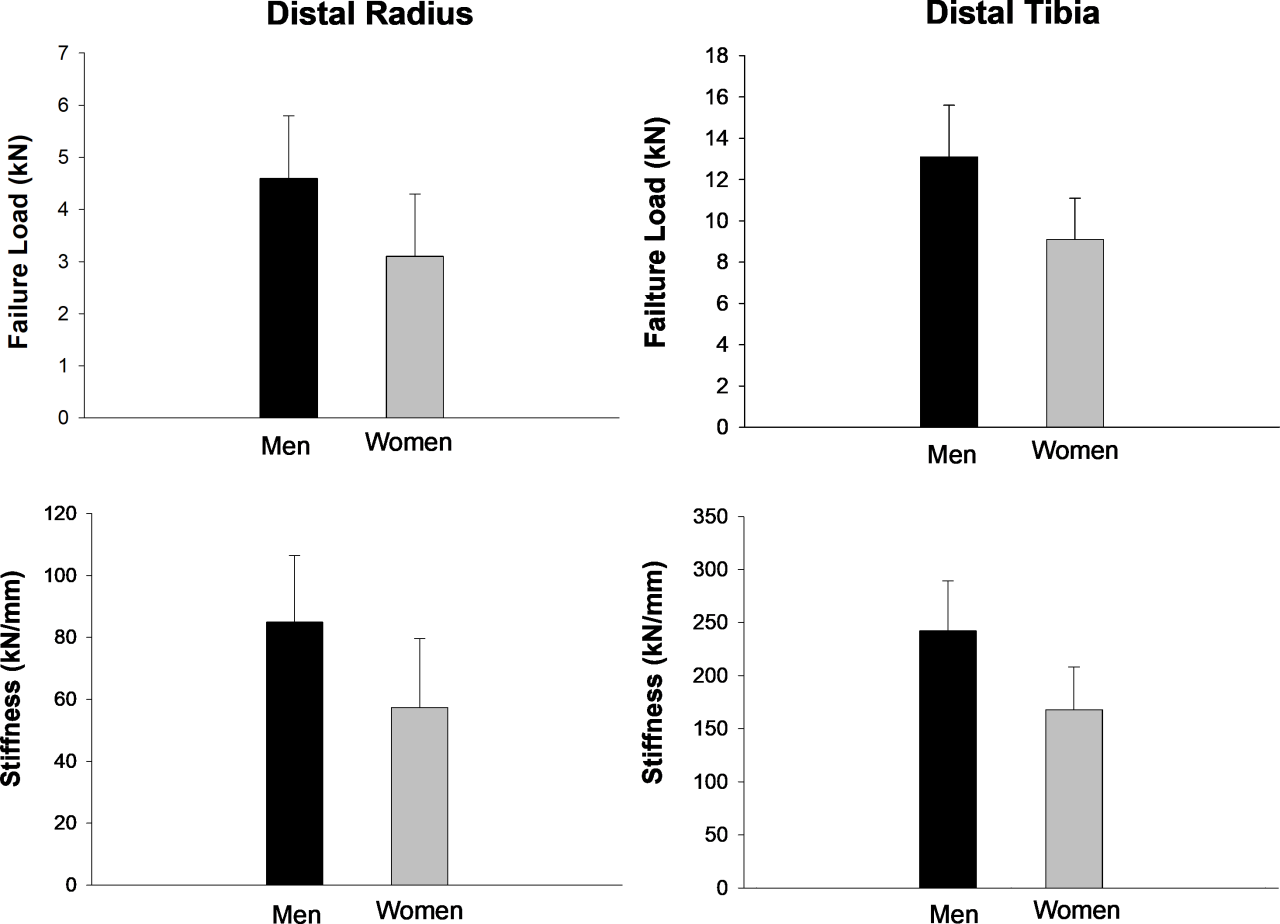
HR-pQCT bone strength characteristics at the distal radius and tibia in the study participants. Values are mean ± SD. All parameters were significantly different between men and women (all *p* < 0.05).

As expected, men had larger whole body and appendicular lean mass than women, while women had larger appendicular fat mass than men (**Table 2**). Men had larger whole body, hip, and spine aBMD than women. Regarding indices of SO, men had larger ALM/W and HGS than women, while women had larger FM% than men. Men had better indices of bone microarchitecture than women (**Table 3**). Importantly, men had greater bone strength based on μFEA of failure load and stiffness at the distal tibia and radius than women (**Figure 1**).

**Table 2:** Body composition, aBMD, and sarcopenic obesity indices in the study participants.

	Men (*n* = 128)	Women (*n* = 61)
Body composition		
Whole body lean mass (kg)	65.0 ± 7.7	46.1 ± 6.2
Whole body fat mass (kg)	44.7 ± 11.0	44.9 ± 9.1
Whole body BMC (kg)	2.7 ± 0.4	2.0 ± 0.4
Appendicular lean mass (kg)	25.2 ± 2.6	19.7 ± 2.6
Appendicular fat mass (kg)	18.9 ± 5.2	21.3 ± 4.7
aBMD		
Whole body aBMD (g/cm^2^)	1.132 ± 0.119	1.024 ± 0.119
Total hip aBMD (g/cm^2^)	1.095 ± 0.151	0.947 ± 0.134
Femoral neck aBMD (g/cm^2^)	0.871 ± 0.142	0.774 ± 0.147
Lumbar spine aBMD (g/cm^2^)	1.212 ± 0.197	1.056 ± 0.155
One-third radius aBMD (g/cm^2^)	0.798 ± 0.073	0.463 ± 0.079
SO indices		
ALM/W	25.2 ± 2.6	19.7 ± 2.6
Percent fat	39.7 ± 4.7	48.7 ± 4.4
HGS (kg)	38.0 ± 8.5	23.3 ± 7.6

Values are mean ± SD. All parameters were significantly different between men and women except for whole body fat mass (all p < 0.05).

aBMD, area bone mineral density; SO, sarcopenic obesity; BMC, bone mineral content; ALM/W, appendicular lean mass/weight; HGS, hand grip strength.

**Table 3:** HR-pQCT bone structure characteristics at the distal radius and tibia in the study participants.

Variable (units)	Distal radius		Distal tibia	
	Men (*n* = 128)	Women (*n* = 61)	Men (*n* = 128)	Women (*n* = 61)
HR-pQCT				
Total area (mm^2^)	381.2 ± 67.0	252.7 ± 62.1	896.7 ± 137.5	678.6 ± 123.8
Total vBMD (mg HA/cm^3^)	313.6 ± 59.5	294.6 ± 64.4	308.6 ± 54.6	281.2 ± 56.0
Tb.vBMD (mg HA/cm^3^)	178.9 ± 34.9	140.8 ± 41.0	187.5 ± 35.2	160.1 ± 37.5
BV/TV (fraction)	0.254 ± 0.051	0.207 ± 0.064	0.276 ± 0.049	0.240 ± 0.051
Tb.N (1/mm)	1.539 ± 0.201	1.373 ± 0.306	1.475 ± 0.279	1.375 ± 0.279
Tb.Sp (mm)	0.621 ± 0.125	0.856 ± 0.802	0.666 ± 0.116	0.741 ± 0.232
Tb.Th (mm)	0.242 ± 0.019	0.226 ± 0.022	0.264 ± 0.026	0.249 ± 0.018
Ct.vBMD (mg HA/cm^3^)	857.3 ± 48.1	839.6 ± 68.3	838.8 ± 60.8	817.3 ± 54.4
Ct.Pm (mm)	85.0 ± 8.5	67.2 ± 8.9	118.2 ± 9.5	101.7 ± 9.2
Ct.Th (mm)	1.106 ± 0.237	0.992 ± 0.216	1.704 ± 0.377	1.455 ± 0.303
CtPo	0.014 ± 0.008	0.012 ± 0.007	0.236 ± 0.031	0.234 ± 0.028

Values are means ± SD. All parameters were significantly different between men and women except for CtPo (all p < 0.05).

HR-pQCT, high-resolution computed tomography; vBMD, volumetric bone mineral density; TbvBMD, trabecular vBMD; BV/TV, bone volume/total volume; Tb.N, trabecular number; Tb.Sp, trabecular separation; CT.vBMD, cortical volumetric bone mineral density; Ct.Pm, cortical perimeter; Ct.Th, cortical thickness; CtPo, cortical porosity.

Each SO index (ALM/W, FM%, and HGS) was associated with bone strength at the distal radius and tibia in bivariate regression analyses (**Table 4**). ALM/W was positively correlated explaining 28% and 36% of the variance in failure load and 28% and 35% of the variance in stiffness at the distal radius and tibia, respectively. HGS was also positively correlated explaining 32% and 36% of the variance in failure load and 32% and 35% of the variance in stiffness at the distal radius and tibia, respectively. In contrast, FM% was negatively correlated explaining 22% and 31% of the variance in failure load and 22% and 30% of the variance in stiffness at the distal radius and tibia, respectively.

**Table 4:** Unadjusted and adjusted associations between sarcopenic obesity indices and HR-pQCT-based bone strength estimated by finite element analyses at the distal radius and tibia in older adults with obesity.

	Unadjusted associations			Adjusted associations^a^		
	*β* (SE)	*p*-value	*R* ^2^	*β* (SE)	*p*-value	*R* ^2^ full model
Distal radius^b^						
Failure load (kN)						
ALM/W	0.529 (0.061)	<0.001	0.280	0.312 (0.085)	<0.001	0.329
Percent fat	−0.473 (0.064)	<0.001	0.224	−0.217 (0.090)	0.011	0.306
HGS (kg)	0.569 (0.062)	<0.001	0.324	0.406 (0.070)	<0.001	0.372
Stiffness (kN/mm)						
ALMI/W	0.527 (0.062)	<0.001	0.277	0.318 (0.086)	<0.001	0.301
Percent fat	−0.470 (0.065)	<0.001	0.221	−0.222(0.086)	0.010	0.299
HGS (kg)	0.567 (0.062)	<0.001	0.321	0.412(0.073)	<0.001	0.346
Distal tibia^b^						
Failure load (kN)						
ALMI/W	0.597 (0.058)	<0.001	0.357	0.307 (0.078)	<0.001	0.448
Percent fat	−0.556 (0.062)	<0.001	0.310	−0.244 (0.076)	0.002	0.432
HGS (kg)	0.602 (0.054)	<0.001	0.362	0.356 (0.073)	<0.001	0.472
Stiffness (kN/mm)						
ALMI/W	0.590 (0.059)	<0.001	0.348	0.308 (0.079)	<0.001	0.434
Percent fat	−0.550 (0.061)	<0.001	0.303	−0.252 (0.078)	0.001	0.422
HGS (kg)	0.592 (0.059)	<0.001	0.350	0.345 (0.073)	<0.001	0.454

HR-pQCT, high-resolution peripheral quantitative computer tomography; ALM/W, appendicular lean mass normalized for weight; HGS, hand grip strength.

a Adjusted for age, sex, race/ethnicity, diabetes, and 25-OH vitamin D.

b One hundred eighty-nine participants were included in the analysis.

Adjustments for potential confounders (age, sex, race/ethnicity, diabetes, and 25-OH vitamin D) accounted for an additional 3% to 8% and 9% to 12% of the variance in the SO indices–bone strength relationships at the distal radius and tibia, respectively (**Table 4**). After adjusting for the confounding variables in separate multivariable regression analyses, the associations between the SO indices and bone strength remained significant with minor changes in *β* values. ALM/W was positively correlated with failure load and stiffness at the distal radius and ulna (*R* ^2 = ^0.301 to 0.448). HGS was also positively correlated with failure load and stiffness at the distal radius and ulna (*R* ^2 = ^0.346 to 0.472). Conversely, FM% was negatively correlated with failure load and stiffness at the distal radius and ulna (*R* ^2 = ^0.299 to 0.432).

Among the 189 participants, 75 (40%) met the criteria for SO, whereas 114 (60%) did not meet the criteria (**Table 5**). Among the 75 participants with SO, 9 (12%) had stage I, while 66 (88%) had stage II. Physical frailty was present in 55/75 (73%) participants with SO and in 66/114 (58%) participants with no SO (*p* = 0.02 for between-group comparisons). Cardiometabolic syndrome was present in 57/75 (76%) participants with SO and in 85/114 (75%) participants with no SO (*p* = 0.48). Importantly, failure load and stiffness at the distal radius were lower in participants with SO vs. those with no SO (both *p* < 0.05) with a trend for also lower failure load and stiffness at the distal tibia. There were no significant differences in aBMD between participants with SO and no SO (**Supplementary Appendix Table 1**).

**Table 5:** Physical and metabolic complications and bone strength as assessed by μFEA of HR-pQCT at the distal radius and ulna in obese older adults with sarcopenic obesity and no sarcopenic obesity.

	Sarcopenic obesity (*n* = 75)	No sarcopenic obesity (*n* = 114)	*p*-value^a^
Physical frailty, *n* (%)	55 (73)	66 (58)	0.03
Cardiometabolic syndrome, *n* (%)	57 (76)	85 (75)	0.48
Distal radius			
Failure load (kN)	71.9 ± 26.9	78.8 ± 26.9	0.048
Stiffness (kN/mm)	3.9 ± 1.2	4.3 ± 1.4	0.04
Distal tibia			
Failure load (kN)	210.9 ± 52.4	223.1 ± 59.1	0.050
Stiffness (kN/mm)	11.4 ± 2.7	12.0 ± 3.1	0.07

Values are means ± SD or n (%).

a Adjusted for age, sex, race/ethnicity, diabetes, and 25-OH vitamin D.

## Discussion

In this population of community-living older adults with obesity, we have shown that indices of SO are significantly associated with bone strength as assessed by μFEA of HR-pQCT at the distal radius and tibia. Specifically, both ALM/W and HGS were positively associated with failure load and stiffness at the distal radius and tibia. On the other hand, FM% was negatively associated with failure load and stiffness at the distal radius and ulna. These associations remained significant after controlling for confounders such as age, sex, race/ethnicity, diabetes, and 25-OH vitamin D. These data suggest that fracture risk in older adults with obesity is likely related to decreased muscle mass and strength, as well as to increased fat mass, which may lead to impaired bone quality.

To the best of our knowledge, the present study is among the first to correlate multiple indices of SO with bone strength as determined by μFEA of HR-pQCT specifically in older adults with obesity. SO is a geriatric syndrome with a multifactorial etiology, characterized by excess fat mass and sarcopenia that negatively influence patient-centered outcomes (7). Although there has been a lack of universally recognized diagnostic criteria, we used indices for diagnosing SO (ALM/W, FM%, HGS) based on the recent expert consensus statement of the ESPEN and EASO (18). Our study providing insights into potential interactions between bone and muscle and adipose tissue is in line with the research priorities proposed in SO (35). In another HR-pQCT study involving participants with a wide range of age and BMI, appendicular lean mass index (ALMI) was also shown to positively correlate with failure load at the distal radius and tibia (36). Because muscle strength was not measured in that study, SO could not be assessed. Moreover, in contrast to the findings in our present study, fat mass index (FMI) was not associated with failure load after adjusting for confounders. Therefore, the results of our present study highlight that, specifically in the population of older adults with obesity, not only lower muscle mass and strength but also higher fat mass contribute to fracture risk because of poorer bone quality. Indeed, μFEA parameters of bone quality derived from HR-pQCT have been shown to be significantly associated with incident and prevalent fractures (25).

The positive association between muscle mass and bone strength can be explained in several ways. One possible explanation for this relationship is the mechanostat hypothesis which explains the adaptation of bone to mechanical loads acting on it (9). Contracting muscles exert mechanical forces on the bone that cause deformations within the tissue. These deformities are sensed by osteocytes that send stimuli for the bone to either maintain or increase its strength. Consequently, greater muscle mass and strength can help to shape the bone structure and increase bone strength. Additionally, skeletal muscle can also affect bone homeostasis in a non-mechanical manner—through its endocrine activity (37). As an endocrine organ, muscle secretes a panel of proteins, called myokines, synthesized and secreted by myocytes in response to muscle contraction. Several of these myokines include growth factors known to stimulate bone formation and strength independent of mechanical loading, thus showing evidence of a muscle–bone relationship (10). On the other hand, the negative association between fat mass and bone strength may be explained by the positive energy balance typical of obesity which worsens the excess fat deposition with aging (38). Accordingly, in older adults with obesity, there is markedly elevated secretion of adipokines by adipose tissues that include proinflammatory cytokines (7). These adipocytokines can stimulate bone resorption and disrupt bone strength by enhancing the activity of mature osteoclasts (11). In addition, some studies suggest a potential role for vitamin D as a common denominator of sarcopenia, obesity, and poor skeletal health in osteosarcopenic obesity (39).

Our findings suggest that lower muscle mass and strength and higher fat mass may compromise bone quality. These findings may help guide clinicians in the treatment of older adults with obesity. Calorie restriction (CR) is effective for reducing fat mass but may also accelerate the age-related decrease in muscle and bone mass (13). Combining CR with aerobic and resistance training may ameliorate adverse effects on muscle and subsequently improve bone quality and decrease fracture risk in older adults with obesity. Previous findings from a 1-year randomized controlled trial in older adults with obesity showed that lean mass and hip BMD decreased less in the combined weight loss–exercise group than in the weight loss group (40).

Because of different definitions and approaches to diagnose SO, prior studies have reported wide ranges of prevalence (e.g., 0.8% to 84%) (7). The present study is the first to determine the prevalence and stage of SO based on the ESPEN and EASO expert consensus criteria in a homogeneous population of older adults with obesity. Our finding that more than a third (40%) of older adults with obesity have SO indicates a subgroup particularly vulnerable to poorer health outcomes due to additive adverse effects of obesity and sarcopenia (41). In fact, among those with SO, the majority (88%) had stage II associated with functional or metabolic complications. In particular, the coexistence of obesity with sarcopenia was associated with a higher prevalence of frailty (73%) and lower bone quality at the distal radius than obesity without sarcopenia (43%). Conversely, SO was not associated with a higher prevalence of cardiometabolic syndrome, probably because of a ceiling effect of obesity in our participants all recruited for excess adiposity.

Our study has limitations. First, the cross-sectional nature of our study precludes the assessment of cause–effect relationships. Second, we used DXA-measured lean mass to assess muscle mass rather than more direct measurements (e.g., D_3_-creatine dilution) which may not be routinely available in clinical practice. Accordingly, the ESPEN and EASO consensus statement endorses DXA as an adequate compromise between the precision and accuracy of measurements and the availability of instruments for clinical implementation of SO diagnosis (18). Third, most of our participants were men, white, and well educated who volunteered to participate in a lifestyle program. Fourth, we did not have data on adipokines and/or proinflammatory cytokines, which would be useful to include in future studies correlating indices of SO with bone quality. Finally, at present, difficulties in the availability of HR-pQCT including costs could potentially limit the impact of its use and derived parameters.

In conclusion, our findings demonstrate that indices used to diagnose SO (i.e., ALM/W, HGS, FM%) may be important determinants of bone quality in older adults with obesity. Therefore, interventions that focus on preserving muscle mass and strength while reducing fat mass may be important to maintain bone quality and decrease fracture risk when older adults with obesity undertake lifestyle intervention therapy.

## Data availability statement

The datasets presented in this article are not readily available because restrictions apply to the availability of some or all data generated or analyzed during this study to preserve patient confidentiality or because they were used under license. The corresponding author will on request detail the restrictions and any conditions under which access to some data may be provided. Requests to access the datasets should be directed to dennis.villareal@bcm.edu.

## Ethics statement

The studies involving humans were approved by Baylor College of Medicine Institutional Review Board. The studies were conducted in accordance with the local legislation and institutional requirements. The participants provided their written informed consent to participate in this study.

## Author contributions

GG: Investigation, Writing – review & editing. AP: Investigation, Supervision, Writing – review & editing, Project administration. YB: Methodology, Supervision, Writing – review & editing, Investigation. AC: Writing – review & editing, Investigation. MS-E: Writing – review & editing, Methodology, Project administration, Supervision. RA-V: Investigation, Writing – review & editing, Methodology, Conceptualization, Supervision. NN: Investigation, Writing – review & editing. CQ: Formal Analysis, Investigation, Methodology, Writing – original draft, Writing – review & editing. DV: Conceptualization, Formal Analysis, Funding acquisition, Investigation, Methodology, Project administration, Supervision, Writing – original draft, Writing – review & editing.
