# Supplementary material for: Indices of sarcopenic obesity are important predictors of finite element analysis-derived bone strength in older adults with obesity
Source: Front Endocrinol (Lausanne). 2023 Nov 7;14:1279321. doi: 10.3389/fendo.2023.1279321 (PMC10660264; doi:10.3389/fendo.2023.1279321)
Supplement: Supplementary file 1 [file Table_1.docx]

**Supplementary Table 1**. Bone quantity (aBMD) as assessed by dual-energy x-ray absorptiometry in obese older adults with sarcopenic obesity and no sarcopenic obesity.

|  | Sarcopenic obesity  (n = 75) | No Sarcopenic obesity  (n = 114) | *p*^a^ value |
| --- | --- | --- | --- |
| aBMD |  |  |  |
| Whole body aBMD (g/cm^2^) | 1.083 ± 0.120 | 1.106 ± 0.135 | 0.20 |
| Total hip aBMD (g/cm^2^) | 1.029 ± 0.152 | 1.059 ± 0.166 | 0.21 |
| Femoral neck aBMD (g/cm^2^) | 0.823 ± 0.147 | 0.852 ± 0.152 | 0.19 |
| Lumbar spine aBMD (g/cm^2^) | 1.158 ± 0.120 | 1.164 ± 0.204 | 0.68 |
| One-third radius aBMD (g/cm^2^) | 0.740 ± 0.095 | 0.761 ± 0.102 | 0.18 |

Values are means ± SD.

^a^adjusted for age, sex, race/ethnicity, diabetes, and 25-oh vitamin d
